
JOURNAL INFORMATION
==============================
NLM Title Abbreviation: ScientificWorldJournal
Journal ID: ScientificWorldJournal
NLM Journal ID: 101131163
Journal ID: TSWJ

The Scientific World Journal

ISSN: 2356-6140
EISSN: 1537-744X
Publisher: Wiley

ARTICLE INFORMATION
==============================
PMCID: PMC6084538
DOI: 10.1100/tsw.2001.433
Article ID: 740137
Article version: 1
Subjects: Extended Abstract

The Role of CGRP in the Development of Morphine Tolerance and Physical Dependence

Powell K. J. 1
Trang T. 1
Quirion R. 2
Jhamandas K. 1
Department of Pharmacology and Toxicology, Queen’s University, Kingston, Ontario, Canada
2 Douglas Hospital Research Centre, McGill University, Verdun, Quebec, Canada, Canada
Publication date: 2001
Electronic publication date: 2001 Feb 9
Volume: 1
First page: 21
Last page: 21
Copyright: Copyright © 2001 K. J. Powell et al.
Copyright year: 2001
License: This is an open access article distributed under the Creative Commons Attribution License, which permits unrestricted use, distribution, and reproduction in any medium, provided the original work is properly cited.
License URL: https://creativecommons.org/licenses/by/3.0/

Funding: Canadian Institutes of Health Research

==============================
4th International Meeting on Calcitonin Gene-Related Peptide (CGRP)
*TheScientificWorld* (2001) 1(S1), 21
ISSN 1532-2246; DOI 10.1100/tsw.2001.433

### THE ROLE OF CGRP IN THE DEVELOPMENT OF MORPHINE TOLERANCE AND PHYSICAL DEPENDENCE

K.J. Powell, T. Trang, R. Quirion[1], and K. Jhamandas
Department of Pharmacology and Toxicology, Queen's University, Kingston, Ontario, Canada;
Douglas Hospital Research Centre, McGill University, Verdun, Quebec, Canada

Chronic morphine treatment produces adaptive changes in spinal neurons that reduce opioid action and lead to the development of morphine tolerance and physical dependence. The spinal neuropeptide calcitonin gene-related peptide (CGRP) has been implicated in these phenomena. We have shown that both spinal and systemic chronic morphine treatment induces an increase in CGRP in the dorsal horn of the spinal cord and that an acute naloxone challenge depletes CGRP from spinal primary afferents. The CGRP receptor antagonist, $CGRP_{8-37}$, blocks the development of tolerance and reverses established tolerance. It also prevents and reverses changes in spinal CGRP expression associated with tolerance. A nonpeptide CGRP receptor antagonist BIBN4096BS was also effective, although less potent than $CGRP_{8-37}$. Additionally, $CGRP_{8-37}$ reduces the behavioural symptoms of morphine withdrawal and prevents the depletion of spinal CGRP during withdrawal. To determine if morphine affects CGRP expression through a direct action on the primary afferent we have recently established an *in vitro* model of cultured adult dorsal root ganglion (DRG) neurons. Here we show that, similar to our *in vivo* studies, chronic morphine increases CGRP expression in DRG cultures and that this increase is completely blocked by $CGRP_{8-37}$. These studies suggest that morphine acts on primary afferent neurons to augment CGRP expression which, in turn, contributes to the development of tolerance and physical dependence. In addition, these data show that CGRP mediates its own expression through activity of presynaptic CGRP receptors. Blockade of CGRP activity may have the potential to inhibit clinical opioid tolerance and physical dependence. [Supported by the Canadian Institutes of Health Research.]
